# Working with a robot in hospital and long-term care homes: staff experience

**DOI:** 10.1186/s12912-024-01983-0

**Published:** 2024-05-08

**Authors:** Lily Haopu Ren, Karen Lok Yi Wong, Joey Wong, Sarah Kleiss, Annette Berndt, Jim Mann, Ali Hussein, Grace Hu, Lily Wong, Ruth Khong, Jason Fu, Nazia Ahmed, Julia Nolte, Lillian Hung

**Affiliations:** 1 Innovation in Dementia and Aging Lab, University of British Columbia, T201-2211 Wesbrook Mall, Vancouver, BC V6T 2B5 Canada; 2 Universitätsmedizin Göttingen, Robert-Koch-Straße 40, 37075 Göttingen, Germany; 3 Department of Communication and Cognition, Tilburg University, Warandelaan 2, Tilburg, 5037 AB The Netherlands

**Keywords:** Formal caregivers, Residents, Patients, Dementia, Institutional care, Implementation science, Technology

## Abstract

Although there is a growing literature on the use of telepresence robots in institutional dementia care settings, limited research focused on the perspectives of frontline staff members who deliver dementia care. Our objective was to understand staff perspectives on using telepresence robots to support residents with dementia and their families. Guided by the Consolidated Framework for Implementation Research, we conducted four focus groups and 11 semi-structured interviews across four long-term care (LTC) homes and one hospital in Canada. We included 22 interdisciplinary staff members (e.g., registered nurses, social workers, occupational therapists, recreational therapists) to understand their experiences with telepresence robots. Thematic analysis identified three key themes: 1) Staff Training and Support; 2) Robot Features; 3) Environmental dynamics for Implementation. Our results underscore the imperative of structural support at micro-, meso- and macro-levels for staff in dementia care settings to effectively implement technology. This study contributes to future research and practice by elucidating factors facilitating staff involvement in technology research, integrating staff voices into technology implementation planning, and devising strategies to provide structural support to staff, care teams, and care homes.

**Supplementary Information:**

The online version contains supplementary material available at 10.1186/s12912-024-01983-0.

## Introduction

The use of technologies as non-pharmacological interventions to enhance the well-being of older adults living in dementia long-term care (LTC) settings is growing [1–3]. Among them, literature concerning telepresence robots have increased in recent years [4, 5]. Telepresence robots could support communication for families when they may not be able to visit residents in person. A key feature of telepresence robots is that families can remotely adjust the height, visuals, audios, and moves of the robot so that they can see residents at a desired angle. In addition, with 3D sensors, the robots can avoid obstacles when navigating the surrounding environment [6]. Robots also were found to enhance the relationship between staff and residents and to add value to care [7].

However, many residents may not know how to use technology for video calls, or may have difficulty holding devices and maintaining eye contact with families on the screen, due to cognitive and physical impairment [8]. According to the Canadian Institute for Health Information [9], 70% of LTC residents experience dementia, and 85% or more experience some form of cognitive impairment. Due to their challenges, these older adults may not be able to implement technologies alone, which would represent lost opportunities and wasted technological resources.

Staff in institutional care/LTC settings are among the likeliest people who help older adults use technology [10]. The support from staff to implement technology-based interventions for dementia care is crucial, and the effectiveness of technologies and the use of robots depend on the staff and their knowledge, so that residents can benefit from the robots [11]. Hence, the voices of staff need to be included in the adoption of telepresence robots. In particular, it is important to include staff input to identify (1) what the needs of residents and staff are, (2) how technology could potentially address these needs, (3) what potential challenges of implementing the technology staff perceive, and (4) what support staff will need to overcome these challenges.

Manley et al. [12] explored the use of telepresence robots in post-acute and LTC medicine by surveying staff. Staff had positive views towards telepresence robots, such as finding the robots in question easy to use and as saving them time on appointments through telemedicine visits. In a study by Niemela et al. [13], staff used a telepresence robot in a 12-week residential care trial to connect older people with their families. However, staff were concerned about the privacy of residents. Koh et al. [14] interviewed healthcare providers and organizational leaders to explore why the latter two implement or do not implement pet robots in dementia care nursing homes in Ireland. Results suggested that some staff perceive robots as too complex to implement. Yuan et al. [7] interviewed aging care staff in Australia to understand their perspectives on using social robots. The care staff referenced both benefits of using such robots (i.e., improving the relationship between staff and residents) and barriers (i.e., difficulty in cleaning robots for infection control). Papadopoulos et al. [15] explored care home workers’ views on using socially assistive humanoid robots in the United Kingdom. The assessed staff expressed mixed views on the robots. Although they thought that robots might supplement their work, they did not feel comfortable having the robots standing beside them and that such discomfort would negatively affect their care for residents. In Australia, Zhao et al. [16] interviewed staff and tech developers about using Information and Communication Technologies (ICTs) to connect residential aging care residents to each other as well as to their families and friends. Many residents could not use the technologies alone and had to rely on staff, which added to the staff’s workload. Also, the necessary infrastructure, such as devices and a stable internet connection, was lacking to support the use of ICTs.

However, among the extant, staff’s perspectives in the implementation of telepresence robot are underexplored. Thus, we present a study to report the facilitators and barriers to implementation of a telepresence robot from the perspectives of staff from different care settings (LTC/hospital/public/private etc.) and professional background (nursing/rehabilitation science/social work etc.) See the description of the telepresence robot in 3.4.

For the present study, we took Collaborative Action Research (CAR) as our approach. Grounded in value-based principles, this methodological approach underscores the transformative potential of research in clinical settings through reflective and participatory practices [17]. Typically, CAR involves cycles of planning, acting, observing and reflecting [17]. By embracing partnership between researchers and clinical staff, the results of early cycles help to reveal relevant issues and priorities to inform subsequent actions for each party and to drive meaningful changes in practice [17]. We believe that CAR is useful and appropriate to engage multiple parties to understand and co-design interventions for complex issues [17], for example, in understanding staff perspective on implementation of robots for residents/patients and family caregivers in institutional care settings.

We adopt the Consolidated Framework for Implementation Research (CFIR) to guide implementation [18, 19]. CFIR suggests enablers and barriers of effective implementation approaches by synthesizing 18 relevant theories, models, and frameworks. It considers five intervention domains: The (1) Innovation Domain refers to the object being implemented (here, the technology, program, service, and policy), while the (2) Inner Setting Domain examines the setting of implementation (here, LTCs and a hospital). The (3) Outer Setting Domain explores the setting outside the care site and the 4) Individuals Domain reviews individuals’ roles and features in the process (here, the roles and skills of staff). Finally, the 5) Implementation Process Domain concerns the strategies and activities used to achieve implementation. Each domain subsumes further constructs, with the CFIR entailing a total of 39 constructs. The CFIR was chosen because it is a framework widely and globally used in the when researching health care implementation practices [14, 20, 21]. For our purposes, the CFIR guided different stages of the study process, including the development of an implementation plan, the creation of interview questions, and our analysis steps, which spanned both inductive and deductive approaches.

## Methods

### Aim

This study aimed to explore staff experiences and views on implementing telepresence robots in LTC and hospital settings. It was guided by the research question, “What are staff perspectives on the facilitators and barriers of implementing telepresence robots in LTC and hospital settings for older adults with dementia?”.

### Design

This study was a part of a larger research project exploring the implementation of telepresence robots in LTC homes, covering five LTC settings in British Columbia [22]. The research team included researchers, trainees, resident (i.e., patient) partners, family partners, and frontline staff champions. Resident partners were older adults with lived experiences of dementia. Family partners were informal caregivers of older adults living with dementia.

We utilized a qualitative approach, collecting data through semi-structured interviews, focus groups, weekly check-ins, field notes and research meetings. This approach yielded rich data and detailed descriptions reflective of real-life occurrences during the implementation process. It enabled the research team gain a deeper understanding of the experiences of staff with telepresence robots at their workplaces [23]. We chronicle this study following the Consolidated Criteria for REporting Qualitative Research (COREQ) (see Appendix 1 for COREQ Checklist).

### Sample and setting

Purposive sampling was used in recruiting staff participants. The eligibility criteria include being employed either full-time or part-time, and actively participating in the implementation of the telepresence robot at their workplace. We intentionally selected key informants including staff who played a central role in the implementation process, such as team leader and experienced frontline staff with significant insight into the challenges and successes of integrating the robot into daily operations. The study settings include four LTC homes and a hospital where the telepresence robots were implemented, three of which are publicly funded. One home is a non-profit and privately funded, and one home is privately funded. Staff ratio differs among the five care homes (see Table 1 for the staffing ratio). The last author LH has relationship established with staff champions from five care sites prior to study commencement. All participants were involved in the care for the resident participants who used the robots in questions as part of a larger study. The frontline staff who worked closely with the project team, such as the clinical nurse educator and social worker, helped identify all staff participants who met the eligibility criteria. They explained the present study to the potential participants and obtained voluntary consent from participants before conducting focus groups and interviews. Some staff participants collaborated with the research team members on project tasks (e.g., training of robot use and coordinating with family participants) before the focus groups or interviews were conducted. We completed four focus groups and 11 one-to-one interviews, either in-person at care site meeting rooms or via virtual (Zoom) meetings, depending on participants’ preferences (see Table 2 for Data Collection Activities in Details). There was not anyone else other than participants present in the in person or virtual data collection. We included a total of *N* = 22 interdisciplinary frontline staff members including registered nurses, social workers, occupational therapists, and recreational therapists (see Table 3 for descriptive characteristics of study participants).

**Table 1:** Staff-resident ratio in five care homes

Name	Funding	Staff-Resident ratio	Remarks
Garden View	Publicly funded LTC Home	Day: 1 staff member to 6 residents Evening: 1 staff member to 20 residents	
Ocean View	Publicly funded LTC Home	Day: 1 staff member to 6 residents Evening: 1 staff member to 8 residents Night: 1 staff member to 25 residents	Across both day and evening, 1 nurse was assigned to every 25 residents. At night, 1 nurse was assigned to every 50 residents
N/A	Publicly funded Hospital	Day: 3 nurses and 4 care aides (7 staff in total to 19 residents) Evening: 3 nurses and 3 care aides (6 staff in total to 19 residents) Night: 2 nurses and 2 care aides (4 staff to 19 residents)	
N/A	Non-profit LTC Home	Day: 1 nurse and 2 to 3 care aides for 16 to 17 residents Evening: 1 nurse and 2 to 3 care aides for 16 to 17 residents Night: 1 staff (nurse or care aid) per care neighbourhood (16–27 residents)	A registered nurse was available 24/7 in the care home
N/A	Privately funded, for-profit LTC Home	Long-term care: 1 Resident Care partner for 5–6 residents	Care partner is equivalent to care aides in other four sites in this study

**Table 2:** Data collection activities in details

Focus Groups	Site	Number of Participants
Focus Group 1	Public Hospital	8
Focus Group 2	Garden View	2
Focus Group 3	Ocean View	2
Focus Group 4	Ocean View	2
Total number of participants from focus groups		14
**One-to-one Interviews**	**Site**	**Number of Participants**
5 Interviews	Public Hospital	5
1 Interview	Garden View	1
1 Interview	Ocean View	1
3 Interviews	Non-profit LTC	3
1 Interview	Private LTC	1
Total number of Interviews/participants from Interviews		11

Remarks: In Garden view, one staff member participated one focus group and one individual interview. In ocean view, one staff member participated two focus groups and one individual interview

**Table 3:** Descriptive characteristics of study participants (*N* = 22)

Characteristics	*n* (%)
Roles	
Activity assistant	1 (5)
Care aids	4 (18)
Clinical operations supervisor	3 (14)
Clinical operations educator	1 (5)
Clinical educator	1 (5)
Life enrichment coordinator	1 (5)
Patient care coordinator	1 (5)
Program assistant	2 (9)
Registered nurse (RN)	3 (14)
Rehabilitation assistants	3 (14)
Social worker	1 (5)
Total care worker (TCW)	1 (5)
Gender (self-report)	
Women	19 (86)
Men	3 (14)
Age group (years)	
20 – 29	6 (27)
30 – 39	5 (23)
40 – 49	10 (45)
50 – 59	1 (5)
Ethnicity	
East Asian	9 (41)
South Asian	3 (14)
Caucasian	5 (23)
Southeast Asian	5 (23)
Type of Care Site	
Hospital	13 (59)
LTC	9 (41)

### Description of the telepresence robot implementation

**Fig. 1:**
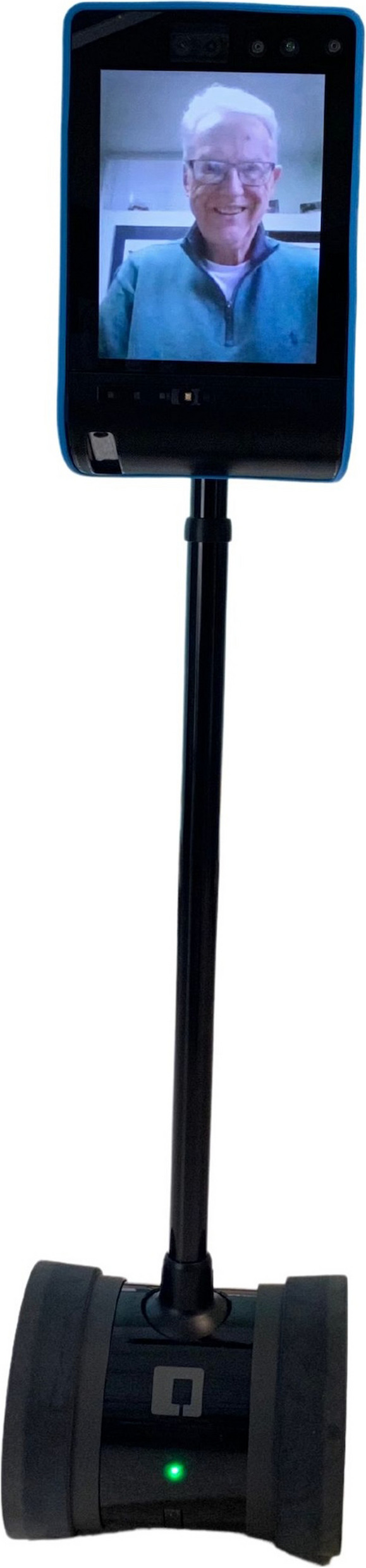
A telepresence robot

The telepresence robot Double (see Fig. 1) was used in our study. The robot has a screen on top which shows the virtual visitor, and wheels at the bottom so that the visitor could drive it to desired locations. Family caregivers had full control of the robot and no efforts were needed from residents to be involved in the robot-facilitated videoconferencing. Although there is an “end call” icon on the robot’s screen, which was designed for residents to have the autonomy to end the call, all the calls were ended by the family members in our study.

The robots were set up at all sites was turned on for use, charged and pre-set to a designated Wi-Fi network all the time. In four care homes, the telepresence robot was placed within residents’ room for a duration of two to 12 months. Some residents were in single rooms, whereas some were in double-occupancy rooms. Family members had 24/7 access to the robot. They could call in to the residents through the robot using an Internet browser or the Double application on their laptops, smartphones, or tablets. In the hospital dementia care unit, the telepresence robot was placed in the nursing station. The staff moved the robot to the residents’ room 10 min before a scheduled family call and moved it back to the nursing station after the call. Before using the telepresence robot for the first time, family members received a 30-min in-person or online orientation by the research team and were provided with family user guides and instructional. Each call between residents and family members lasted between two and 50 min, depends on their preferences. Residents and family members of diverse cultural backgrounds, functional capacities, and gender were recruited purposively to engage with this robot. Some of the resident robot users were more fluent in verbal expression, whereas others had communication and cognitive difficulties in social interactions.

Training for staff at each site involved 20 to 30-min in-person or virtual (Zoom) sessions. The periodic training included demonstrations of the robot uses, safety remarks, and interactive games. Our research team created staff packages containing information about the study and the robot; materials, including educational posters, training videos (see Appendix 2a and b), and newsletters, were distributed to the staff. Staff were asked to assist with cleaning and emergency assistance regarding the telepresence robots, such as keeping the robots charged and connected to the Internet. The research team worked closely with the staff and family members to decide on the best location for robot placement. The research team also had regular check-ins with the staff online or in-person to address their concerns and answer their questions.

### Data collection

Data collection occurred between June 2022 and July 2023 via individual interviews, focus groups, monthly meeting notes, regular staff check-ins, weekly and monthly meetings, observations, field notes, and internal research team reflections. The one-to-one interviews were conducted when focus groups were impossible to carry out at the site (e.g., the site did not have enough staff to deliver care if more than two staff members were participating in an interview at once). One-to-one interviews ranged from 15 to 45 min. The number of participants in focus groups involved two to eight staff members, with each focus group meeting ranging in length from 20 to 45 min. The research team performed weekly check-ins (depending on the availability of staff) to collect updates from the sites. For in-person check-ins, the research team visited the staff champion and staff members at the time and location of their convenience. Fieldnotes were taken during staff check-ins.

Weekly meetings lasted for 30 min, with attendees of a person living with dementia (patient partner), a family partner and trainees including those who were staff champions from the research team. The monthly meetings were 60 min, held online or in-person. Patient partners, family partners, and more staff champions were engaged in discussion on the robot implementation during these meetings. Frontline staff shared their experiences with adopting the robots (e.g., challenges and strategies to overcome these challenges) in regular meetings and check-ins. Through weekly and monthly meetings, the interviewers’ assumptions have been consistently addressed and challenged by multiple partners. The Principal Investigator LH guided meeting discussions.

Data collection ceased upon reaching a point of information saturation, where sufficient data had been obtained to adequately address the research question. The sample size was determined by information power, taking into account factors such as the study's aim, the richness of dialogue, and the specificity of the sample [23, 24]. Interviews were conducted by the authors LR, JW, NA, and GH. LR, JW, and NA are female master graduate student trainees, and GH is a female undergraduate student trainee. Interviewers received training on conducting qualitative interviews and were supervised by LH, the last author who is a female researcher.

The interview guide (see Table 4) consisted of three questions that covered the topics of general staff experiences and of facilitators and barriers to implementing telepresence robots in the workplace. The interview guide was developed by the Principal Investigator LH and refined based on the discussion with the patient partner JM. The interviews were audio-recorded with participants’ consent and transcribed verbatim by trainees. Field notes were documented for additional reflexive thoughts by interviewers. Transcripts were not returned to staff participants for review as they were busy.

**Table 4:** Interview guide

**#**	Questions
1	What is your opinion and perspective on using the telepresence robot in your workplace after seeing others using it?
2	What will enable and hinder you from implementing the telepresence robot in your workplace?
3	What resources, in your opinion, are needed for successful implementation?

### Data analysis

A thematic analysis was performed based on guidelines by Braun and Clarke [25]. This entailed six steps: (1) Three authors (LR, GH and NA) read the transcripts and fieldnotes several times and familiarized themselves with the data, following which (2) LR generated initial codes and the academic supervisor (LH) reviewed the codes. Then, (3) initial themes were generated based on the codes and extracted data, so that (4) the whole team could discuss them. In a next step, (5) the whole team collectively refined and named the themes, and the first author selected quotations for writing the manuscript (see Table 5 for an example of the thematic analysis). Finally, 6) the last author guided the student authors (LR, JW, KW, SK GH, AH, RK, JF and NA) in writing the first draft of the manuscript. All authors reviewed, edited, and agreed on the final manuscript. The authors analysed the data manually on paper-based transcripts.

**Table 5:** Example of the thematic analysis

Quotations	Code	Subthemes	Themes
“Initially we had fear about the robot. After your training, we understand it more.”	Changes in feelings and attitude towards the robot	Addressing staff concerns	Appropriate staff engagement and training
“Staff members can just put it in a room, which gives a family member a window of time to call in.”	Minimum involvement in the family-resident connection	Enables communication that is independent and real-time	Convenient and user-friendly features

### Ethical considerations

Ethical approval and permission for the study were received from the local University and Health Authority (Ethics ID: H22-00659). All staff participants provided written consent. To protect the identities of staff participants, residents, and families, pseudonyms were used in this article.

## Results

Three themes emerged, based on the most imperative facilitators and barriers identified. Themes, facilitators, and barriers are indicated in Table 6, including the acronym of our facilitators ACE.

**Table 6:** Themes, facilitators and barriers

	Theme 1: Staff training and support	Theme 2: Robot features	Theme 3: Environmental Dynamics
Facilitators (ACE)	**A**ppropriate training and support to staff 1) Addressing staff’s concerns 2) Self-motivated staff members 3) Enjoyable and rewarding experiences	**C**onvenient and user-friendly features 1) Enables independent and real-time communication 2) Simple operation 3) Advantages over other ICTs	Resourceful **E**nvironments 1) Supportive leadership 2) Effective teamwork 3) Established family-staff relationship 4) Family autonomy
Barriers	Constraints in training 1) Inadequate internal communication about the robot 2) Limited in-person training 3) Meeting diverse individual learning needs	Non-customized design 1) Heavy weight 2) Low volume 3) Small screen 4) Challenges in charging	Insufficient resources and structural supports 1) Wi-Fi issues 2) Challenges in human resources 3) Lack of technical support 4) Limited physical space

### Theme 1: Staff training and support

Staff highlighted how training and support with different characteristics facilitate or hinder them from understanding and using the robots with different effects. The training and support were mainly from two sources: the research team and the self-motivated staff members.

#### Facilitators: Appropriate training and support to staff (ACE)

Staff mentioned what kind of training was considered of quality, was conductive to supporting their understanding and use of robot, and was effective in addressing their concerns towards the robots. Staff also elaborated how enjoyable and rewarding experiences gained from receiving training and supports motivated them to use the robot more.

##### Addressing staff’s concerns

“After your training, we understand it more.”

Staff highlighted the impact of training on their attitude towards the robot per se and its implementation upon initial introduction of the robot.

Emily (RN): *“Before the training, I have not touched it [the robot] because I am not sure. I was hesitant.”*

Pierre (RN): *“You don’t want to mess it up or something.”* (laugh)

Emily (RN): *“But today, I feel better. More comfortable.”*

Pierre (RN): *“After your training, we understand it more.”*

Emily (RN): *“It [the training] stays more in your head with the symbols. Stays more in your brain.”* (Focus Group, Public Hospital)

*“Initially we had fear about the robot... there was a lot of fear. Like, am I going to be caught doing something that the family's not going to be happy with or? Is the family able to look on (through the robot) and then make complaints what they don't like? Those were some of the concerns that I think we addressed. I think your team has done a very good job of addressing our concerns on technology and privacy for both other residents and staff. I think staff education and the actual demonstration of how to use the robot helps. I think our staff has moved past that (concerns).”* (Bridgette, Clinical Operations Supervisor, Garden View)

As staff started using the robot, provision of ongoing support was as important as the initial orientation.

*“I remember the first couple of sessions, all staff were not sure [how to use the robot]. But the last [training] session I was involved in, I was doing my thing here, just listening in and you got people talking and involved. It (the training) was very informative, and people were really engaged into it…Over the time you have been here to show the staff how easy it is…just getting the staff really comfortable with it.”* (Ashley, Patient Care Coordinator, Public Hospital)

##### Self-motivated staff members

*“I look for ways to use the robot.”*

*“I learned from our staff champion, and it worked brilliantly.”*

Staff champions who are proactive and self-motivated in using the robot were positive influences on the larger team. A staff champion at a private LTC home described how her implementation of the robot influenced her colleagues and helped the care team to start using robots.

*“I look for ways to use the robot even outside of scheduled calls. When I put the laptop to be set up on the ground floor where entertainers are, residents and staff ask me, ‘What is that?’ And then I explained how people on the second floor cannot come downstairs can still attend the performance through this robot. Also, I brought the robot back and forth, which is important as it serves as a good visual reminder for other staff that this is something helpful for the residents. It can cause the other staff maybe to be motivated to engage as well. Staff can take it in by observing, and then from there, it might encourage their willingness to participate as well.”* (Natasha, Life Enrichment Coordinator, Private LTC home)

A passionate staff champion could also disseminate knowledge about the robot by integrating this innovation into the new staff’s orientation and contributing to adoption of the robot by their new colleagues. Participants from the public hospital described how their staff champion Frank introduced the robot to the new members.

*“Frank is really good. We have a new staff [member], I know he has talked about it (the robot) and showed her. He is a great team member. Very open to sharing things like that. We are very lucky to have Frank on the unit. He's very helpful.”* (Ashley, Patient Care Coordinator, Public Hospital)

*“I did two orientation days with Frank and he introduced me to the robot. He showed me how to make sure it was parked properly in the charger, how to set up the visitors pass, how it worked on the family members’ end, and so on.”* (Jamie, Rehabilitation Assistant, Public Hospital)

It is also important to have staff members who are willing to learn from the staff champion (e.g., via peer influence). Julia mentioned how she initiated learning from Frank:

*“I am one of those apprehensive people with technology. The robot was all new to me. I remembered when Sandra (a daughter of a resident) first started [using] the technology, Frank was going to give Sandra the orientation and show her how to move the robot within the room. So I said to Frank, ‘Hey, can I be present with you?’, and I learned from him. That's what we did, and it worked brilliantly.”* (Julia, Social Worker, Public Hospital)

##### Enjoyable and rewarding experiences

*“Once staff know that there is something for them to gain, they are more into it.”*

In our observation, regular check-ins, and meetings, our research team noticed that the frontline staff and team leads from the five care sites were often overloaded. To incentivize them and lighten their workload, refreshments (usually bubble tea) and the latest newsletters were provided before each education session. Staff became more relaxed and attentive, with a curiosity to learn about the robot training. Natasha mentioned, “*They (staff) really like the bubble tea and the training with that.”* (Natasha, Life Enrichment Coordinator, Private LTC Homes).

**Fig. 2:**
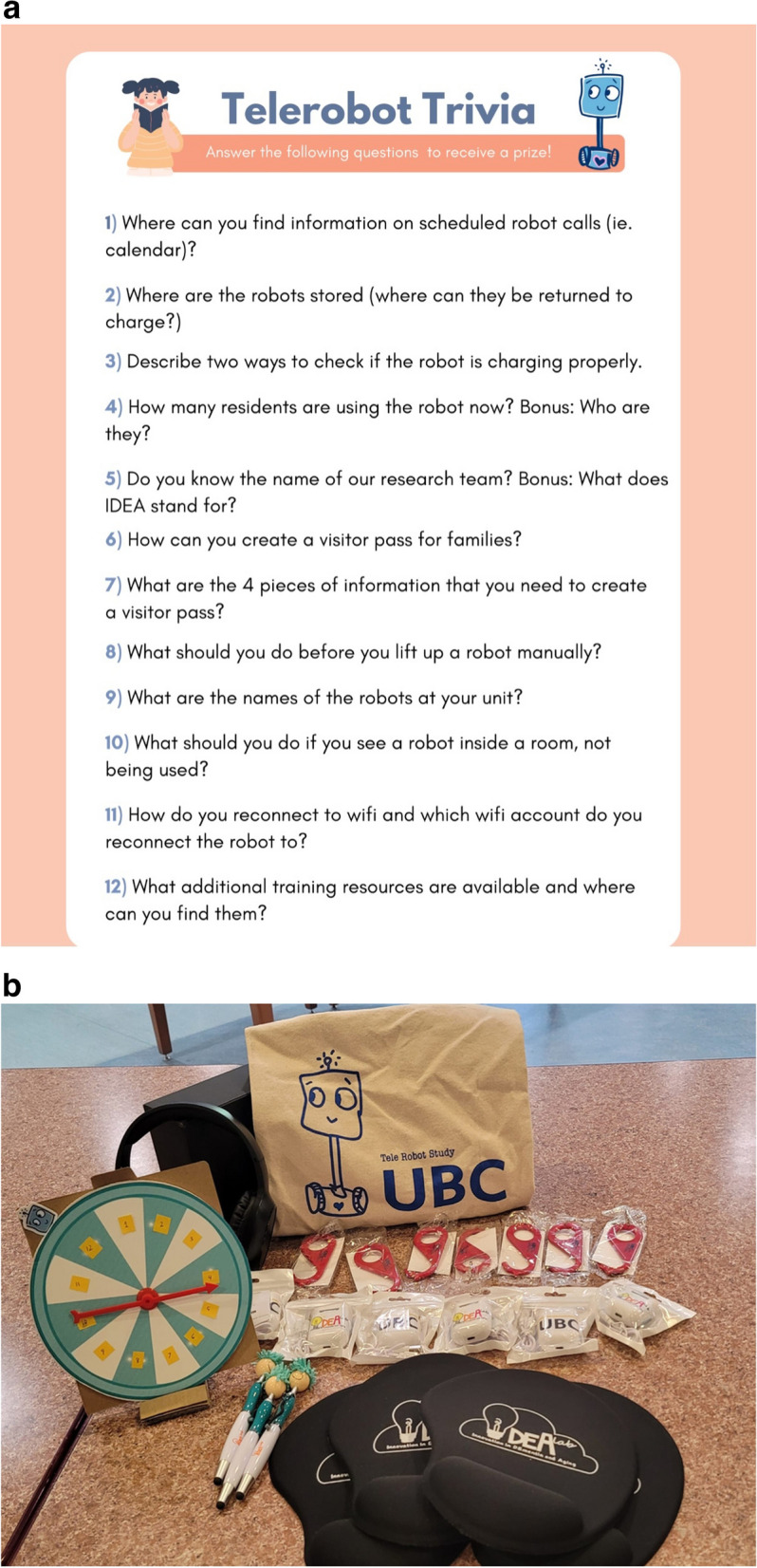
**a** Trivia questions for spinning wheel. **b** Tools for staff training

Gamification was applied during robot demonstrations/trainings. Before each training, tools such as spinning wheels, trivia lists, and prizes (i.e., earphones, keychains, tote bags) for staff were prepared, as Fig. 2a and b show. Each staff member was invited to spin the wheel after the introduction. When the wheel stopped at a number between 1 and 12, the staff member was asked a corresponding question from the trivia list. The staff member, or the player, who answered the question right was awarded a prize and cheered on by the entire care team and research team. Staff members who did not answer the question right were also rewarded with gifts from the research team during the session.

A team lead who observed her team members during the training explained how gamification worked well in engaging them:

“*Your training is good. The spin wheel is great because your little prizes just get people engaged. Once people know that there is something [for them] to gain, even though it is little, they are more into it. Definitely.”* (Ashley, Patient Care Coordinator, Public Hospital)

In addition to attending engaging training sessions, staff also reported positive experiences generated from using the robot and successfully enabling communication between residents and families. For instance, staff talked about how using the robot in supporting the resident-family connection made them feel their work is meaningful.

*“I saw the delight in Sandra (a daughter of a resident)’s eyes when she figured out how to operate the robot [and virtually visit her father here]. At first, she had a bit of trouble, and it (the robot) went to the wrong way. Then Sandra operated it correctly, and she had it going right down the hallway, and you can see the delight in her eyes on the screen showed ‘Wow! This is actually working, and I can do it remotely from Europe!’ It was pretty cool. The robot made those meaningful moments of joy and happiness. That makes my day.“* (Julia, Social Worker, Public Hospital)

*“The robots are very useful – they bring a positive influence to the residents' lives. The research brings lots of happiness to the residents. I appreciate your research.”* (Amanda, Activity Assistant, Villa Cathay)

*“If the family can use it properly and the resident is able to engage, I feel happy and content about helping the residents and being part of the project.”* (Courtney, Clinical Operations Supervisor, Ocean View)

**Fig. 3:**
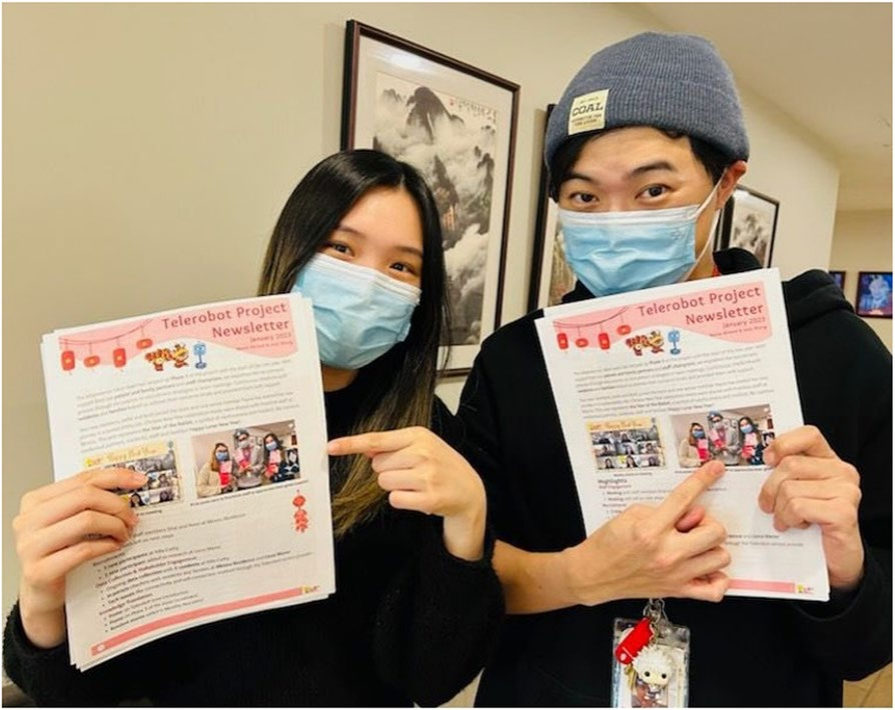
Staff were excited to see themselves in the newsletter

Staff also gained positive experiences by seeing the knowledge translation outputs developed by the research team that acknowledged their active usage of the robot. For example, a newsletter for the project was issued every month and disseminated among staff from all care sites. Staff felt rewarded by seeing themselves and their successful stories of using the robots featured in these newsletters (as Fig. 3 shows).

*“I am ok with the February newsletter because I am here (Alex pointed himself on the newsletter). Do you see me here? (laugh)”* (Alex, Clinical Operations Educator, Ocean View)

Natasha, the Life Enrichment Coordinator from the private LTC home, shared: “*From (my) experience, the staff got excited when they saw their own pictures. Also, I think those special events that you did, for example, for Mother's Day when you brought the flowers, that was really impactful for the staff. It caused a positive link between the robot and just feeling appreciated for being there to support.*” She further suggested that future engagement could be *“combined with maybe a special event*… *This way you can show some appreciation, but also refresh them (the staff) on the use of the robot.”*

#### Barriers: Constraints in training

Staff pointed out factors from the internal and external environment hindering their learning, as well as their diverse learning needs. This includes limited information sharing within the care team about the robot, insufficient in-person training and different learning preferences of each staff member.

##### Inadequate internal communication about the robot

Staff described how poor knowledge dissemination about the robot and its implementation within the team resulted in limited robot usage.

*“I think 80% of the staff members do not know how it (the robot) works. Usually it is just us [program assistants] that are in charge of this robot [who know it]. I think the nurses and care aides upstairs don’t know how to use it.”* (Lora, Family Visitation Program Assistants, Non-Profit LTC)

Bridgette reported a similar situation: *“We have our rotations, so it is not the same people every time you come. I know we have done some training with staff, but they may not be working that day. Definitely a matter of training.”* (Bridgette, Clinical Operations Educator, Garden View).

Moreover, the focus group at the Ocean View, the public LTC home elucidated that reliance solely on self-motivated individuals (often staff champions) generates limited influence on the others in the team in using robots.

*“Sarah (Clinical Educator) has been excellent, she goes and checks. When I am here, I go and check. But on the weekends and after-hours, we are not here. If our staff find it overwhelming, they will probably not know what to do.”* (Courtney, Clinical Operations Supervisor, Ocean View)

##### Limited in-person training

The implementation of robots was started during the COVID-19 pandemic, when significant restrictions on in-person visitation were placed on LTC homes and hospitals [26]. As a result, the care team could only access the robot-related training and support online during that time period. One of our staff participants explained why in-person interaction is preferred over virtual connection:

*“I find that in-person interactions are the most meaningful. Being in person is such a different relationship and feeling. You feel more connected. But because of the pandemic, you were not able to come and do in-person training or education. There were a lot of emails and phone calls back and forth when they were using robots. I think that was definitely one of the barriers – probably one of the biggest barriers.”* (Julia, Social Worker, Public Hospital)

As the in-person visitation limitations lifted in 2022 and in-person training and support could be provided again, Ashley shared her view on those in-person training sessions that her care team received: *“When you came in to do those information sessions, I saw from the last couple sessions, you got the team very engaged. They're very happy to see you. It (the in-person training session) helps.”* (Ashely, Patient Care Coordinator, Public Hospital).

##### Meeting diverse individual learning needs

Staff spoke of their diverse learning needs, given variations in individual roles and routines. For instance, Jasmin in the focus group mentioned the need for printouts for her to learn about using robots in addition to some in-person trainings:

*“You have been doing in-person trainings but we also like printouts…I know there is already something there. But there could be also a binder that I came across with information in it, like the signage there. So I guess the mixture of resources is good. You guys have already been helping us with that.”* (Jasmin, Total Care Worker, Public Hospital)

Julia explained why some of our existing training materials were not tailored for frontline staff members: *“Some of us, like physicians, we check our emails regularly and do Zoom meetings and connections. For nurses and TCWs (Total Care Workers) who provide direct care, they are not always on their email throughout the day. That (virtual communication) is not their norm. If you just send them emails, packages, or a video on how the robot works, you are not getting the same buy-in or interest, because they don't feel involved. For them, in-person orientation is better, making them feel invited and included.”*( Julia, Social Worker, Public Hospital).

Interestingly, Lora expressed learning-related preferences that differed from the abovementioned formats: *“It is better to have an overview, like an orientation about the robot. For example, how to create a password for the family members and how to teach them to use it. Guidelines on how to teach a family member on how to use their phones to control the robot would be great. A manual or both staff and family members would be enough. A YouTube video or[other] video would be better than a book as a guide.”* (Lora, Program Assistant, Non-profit LTC home).

### Robot features

Staff reported how features in the telepresence robots facilitate or hinder implementation, depending on the care team, the environment of the care sites and implementation scenarios of communication and operation.

#### Facilitators: convenience and user-friendly features (ACE)

##### Enables independent and real-time communication

“It is a very valuable tool.”

Staff described key features that made the robot easy for them to use and to support their residents or residents’ social connections. Firstly, the most prominent strength was enabling independent, direct, and live videoconferencing between the residents and their families. Some participants described how little staff involvement was needed in the virtual visitation of families to residents.

*“I think one of the biggest benefits to the robot is that staff members can just put it in a room, which gives a family member a window of time to call in. If everything is running properly, it (the robot) takes me only 30 seconds out of my day in the morning. I put the robot in the room and I can move on to my other tasks.”* (Jamie, Rehabilitation Assistant, Public Hospital)

*“I just need to send the link, put the robot in resident’s room and put back in the dock (after the videoconferencing). Other staff have similar positive experiences. They said it is easy.”* (Frank Rehabilitation Assistant, Public Hospital)

Similar experiences were reported by staff from the public LTC home Garden View:

*“I do see this [robot] as a very valuable tool – for families to have the ability to connect on their own without having someone setting it up for them (i.e., Skype or iPad that we had to set up). The families can do it whenever they want. All they have to do is to call us and ask us, ‘Can you send us the link as we have our loved one in the room?’”* (Lisa, Clinical Operations Supervisor, Garden View)

Secondly, staff members also explained how the independence in the resident-family communication facilitated by the robot freed staff from being perceived as an “agent:”

*“[The robot] is not hindering the workflow of staff. It is more compatible with our workflows because the family can connect directly to the resident without thinking [of] us as the middle people.”* (Bridgette, Clinical Operations Supervisor, Garden View)

*“It (the robot) makes a big difference. It lessens the workload. Because the families can directly call in [to] the residents.”* (Alex, Clinical Operations Educator, Ocean View)

Lastly, for real-time communications, one of our participants recalled how the robot helped the visitor (a daughter) to address the resident (mother)’s mental well-being instantly during the virtual visitation.

*“Whenever they called, the daughter reads her mother’s facial expressions depending on how she talked, for example if the daughter was too loud, or if she said something that confused her mom. The daughter could immediately see whether she needs to adjust her wording or to lower her volume based on her mom's live facial expression. Those little gestures could be captured by the robot and help her mother understand better and not feel frustrated.”* (Natasha, Program Assistance, Private LTC)

Another story shared by staff indicated how the robot’s real-time communication function enabled family members to virtually accompany a resident during therapy:

*“Therapists came to our residents for acupuncture. The family members wanted to see what they (therapists) are doing, to soothe them (the residents), and to translate between the residents speaking Cantonese and therapists speaking English. That is why we put the robot in the room and let them (families and therapists) communicate. For example, the residents can say where the pain is and which procedure makes them feel better, and the families translate between the residents and therapists, then the therapists could proceed more effectively. In that way, the session went well and became more effective.”* (Lora, Program Assistant, Non-profit LTC home)

##### Simple operation

The second biggest facilitator that participants highlighted was the simple operation of the robot; this made it easier to set up calls for residents, teach residents’ families how to use it, and innovatively apply robot usage to novel situations. Consequently, most of the staff members found that having this new technology in their daily routine did not impact their workload, and instead even reduced their it.

*“I just follow the steps, since it is easy to follow – click the link and send it. Very straightforward. I think it is so good, especially given the increasing demand for the health care professionals. The workload for us is very heavy. And this [robot] helps them (staff) to still give support to the family and patient, while not overworking or increasing workload. I think staff would benefit having a robot. It’s awesome.”* (Francisca, Rehabilitation Assistant, Hospital)

In our focus group in the hospital, the multidisciplinary care team told us that:

*“I like that it (the robot) is very user-friendly. Only four icons – easy enough to use.”*

*“Within 5 minutes you can set it (the robot) up with a family member … Workload-wise, it is not affecting us.”*

*“[Tablets] are charged more frequently. The robot could [be used] for 3 hours.”*

*“The QR code makes it much simpler for you to use [the robot than a tablet]. The other one (tablet) you have to wait and punch in the number…all that kind of stuff….it (the tablet) looked [a] little more complicated to me.”*

Similar experiences were also reported by staff from the Non-profit LTC home:

*“I just put the robot in the resident's room, scan the QR code, enter the duration [of the link], copy the link, and send it to the resident's family member. Then I can teach them how to use it (the robot) so it is quite easy for me.”* (Lora, Program Assistant, Non-profit LTC home)

##### Advantages over other ICTs

In terms of easiness and difference in experience, the staff also compared the robot with other devices or software that they have been using.

*“It is very easy. It is easier than, say, FaceTime, because [with the robot], you just need to email a link [to the families] and then they just connect. It is easy at both ends. You just need the other person's email. The robot does not show any information about where it (the email) came from because it's all private. It is comfortable to use it because it is so simple. It really, literally, is so simple.”* (Ashley, Patient Care Coordinator, Hospital)

*“I find the robot is more helpful [than a tablet]. Because otherwise we have to grab a tablet, and you have to log them (the families) on.”* (Bridgette, Clinical Operations Supervisor, Ocean View)

#### Barriers: non-customized design

Staff reported some features in the robot failed to address their needs at work and older adults’ needs for communication, which discouraged staff from using robots.

##### Heavy weight

The robot weights 7.3 kg, or 16 pounds [27] and two female staff members reported that for them, the robot is *“too heavy to carry:” “There are only two robots in the facility, so the staff has to move them [with their hands] and place in the residents’ room.”* (Amanda, Activity Assistant; Jane, Program Assistant, Non-profit LTC home). The research team assigned three more robots to the care home after the interview.

Other than manually carrying the robot, theoretically, staff have another option to move the robot, which is to drive the robot to the desired place, for example, the room of the next resident who is using the robot. However, in our regular check-in at the site, staff told us that it is generally time-consuming for the robot to be connected or re-connected to the desired Wi-Fi. Hence, it was faster to manually carry the robot to the destination given the busy schedule and heavy workload of staff.

##### Low volume

Staff also spoke of the too-low volume of the robot for residents with hearing challenges, which did not help to improve the residents’ experiences. For example, the private LTC home that Natasha worked at once hosted a performance by entertainers on the site’s first floor, but some residents with mobility challenges had to remain on the site’s second floor. Natasha described her experiences with the low volume of the robot after setting it up for residents who could not experience the performance from the first floor:

*“I put the laptop next to the entertainers, and placed the robot next to residents. The volume [of the robot] is quite small or quite low, even though at its maximum. You can barely hear the music downstairs – it (the robot) was quiet. So I stopped doing that on the second floor because it was a little bit too quiet for residents to hear. I hope can still ‘attend’ the performance through this robot [in the future].”* (Natasha, Life Enrichment Coordinator, Private LTC home)

Two participants also reported similar observations:

“*Some of our residents are really old and cannot really hear from the robot, which is too quiet for them.”* (Amanda, Activity Assistant, Non-profit LTC home).

*“It would also help if the robot’s volume can be amplified based on the person's hearing needs.”* (Courtney, Clinical Operations Supervisor, Ocean View, public LTC home).

##### Small screen

Staff expressed concerns that the size of the screen (9.7 inches) [27] could be too small for residents with visual impairments. For example, one staffer mentioned: *“Some residents have really bad eyesight and do not use glasses. It would be beneficial to have the option to enlarge the screen.”* (Amanda, Activity Assistant, Non-profit LTC home).

##### Challenges in charging

Charging the robot is a crucial barrier expressed by staff member Lisa, who suggested that challenges such as charging require more staff input. *“The families are having difficulty driving the robot back to the dock. If [the robot was] not docked back, it was often not charged. When they want to use it the next time, it has no power. That was when we get the staff to help and assist.”* (Lisa, Clinical Operations Supervisor, Garden View).

### Theme 3: Environmental dynamics

We identified environmental factors that support or undermine staff’s usage of robot across different types of care homes, spanning physical, infrastructural, organizational, relational and cultural environment in our partnered care sites.

#### Facilitators: Resourceful environment (ACE)

Though the environment in each care site is unique, our findings showed four resources and strengths as enablers of robot implementation in care sites: supportive leadership, effective teamwork between staff members, established family-staff relationship and family autonomies in using the robot given by the care team.

##### Supportive leadership

The leaders explained how implementing the robots supported the care teams’ care delivery, especially with respect to connecting residents and families directly and with respect to supporting the care team in achieving its goals:

*“The main driving factor for me [to use the robot] is that it connects [the residents] with the family, despite them not being on-site, meanwhile without causing too much stress on the staff because it was already a very stressful time for everybody.”* (Courtney, Clinical Operations Supervisor, Ocean View)

*“It started within two weeks as I was watching staff deal with the robot with ease. I've seen quite a few instances where it was really benefiting the residents, especially Johnny. He has expressed that he does appreciate it when he has those [robot] calls.”* (Alex, Clinical Operations Educator, Ocean View).

*“I think it (the robot) is another great piece of equipment that can make life nicer while patients in here (the hospital) are separated from family. So, whatever we can do to make connections, continue with family members, friends, whatever it is, should be made available and widely used. I fully support it (the robot). It's good.”* (Ashley, Patient Care Coordinator, Public hospital)

A staff member mentioned how care team leaders’ buy-in eases the integration of the robot into care plans supports the robot’s maintenance:

*“When I initially started having the robot, some leaders were curious. They asked me, ‘What time are you going to do the call?’ Then they would try to make it to the call. One of the leaders was there when the call is being set up. Later, I asked another leader if she can let the care partners know [about the robot], she agreed and suggested placing it in the care plan. After that, I never had any more issues with robots being not charged.”* (Natasha, Life Enrichment Coordinator, Private LTC home).

##### Effective teamwork

Besides leaders’ buy-in, staff also mentioned how good teamwork enabled seamless access to the robot for residents. For example, staff from the public hospital and the private LTC home showed how they helped each other to facilitate the residents’ connection with their families through the robot, regardless of their heavy workload:

*“Last Friday I forgot to do it (put the robot in the resident’s room). The other staff scanned the robot, sent the email and put the robot inside resident’s room… We have a patient’s wife [who] usually calls between 4 and 7 o’clock. I put it there [at 4pm] and other nurses just need to put it back to the dock at 7pm.”* (Frank, Rehabilitation Assistant, Public Hospital)

*“For anyone [who] wants to call in the evening or in the weekends, when Frank is not there, it is important that we have everyone on board. Overall, for the scheduled calls, the care aides know about the robot, how it works and where to find it, if they were asked to put in someone's room.”*(Jamie, Rehabilitation Assistant, Public Hospital)

*“I would usually always be there [for family calls] for the first 10 minutes, then I leave the room and they would continue their conversation. [After the call,] one of the staff would put it (the robot) back in its place.”* (Natasha, Life Enrichment Coordinator, Private LTC home)

##### Established family-staff relationship

For a care home that has established a healthy relationship between family and staff, a robot is viewed to bridge the positive communication and maintained the mutual respect between staff and families. The three cases reported by Life Enrichment Coordinator Natasha (private LTC home) serve as examples:

*“I was with a resident who was calling her family in their room, and a care partner wanted to check in on the resident. I would invite the staff to say hi to the resident’s family. The family is usually very grateful to the care partner for the care that they provide. All the time that has happened, the family directly thanks them (the staff) for all the care that they've been giving to their family, and they (the staff) really appreciate it. That connection through the robot is really nice, and usually it is not planned.”*

*“One of the residents, Alexandra, recently passed away suddenly because she had a fracture. During her recovery from the fracture, she had calls with her family [through the robot]. [After her passing,] her daughter reached out to me and told me it was a blood clot that got into Alexandra’s lung - that was why she passed away over the weekend. The daughter was really grateful that they had those robot calls, because up until Alexandra moved into our care home, the daughter has not been able to visit. If there was not a robot, they would have had a lot fewer face-to-face conversations. The daughter was really grateful to use the robot. She also told me over email that when she was speaking to some staff over the past couple of days, they told her Alexandra quite enjoyed the calls (over the robot).”*

Further, the healthy family-staff relationship created an open environment and made families feel safe to speak about their timing and needs in virtual visitation with residents. Such relationship facilitated the staff’s application of the robot.

*“The only challenge I heard about was if the daughter is calling [the resident] when the staff is giving care, then they (the family members) don't want to disrupt. Then the daughter was concerned about disrupting staff routine, so she told me it would be nice if she calls more spontaneously instead of scheduling calls. This is why another staff champion brought a robot over and put it in the resident’s room. The challenge was the daughter wanted more spontaneity and flexibility in the timing of her calls. That was solved by having the robot in the resident’s room.”*

##### Family autonomy

Staff from three different care homes noted the importance of family autonomy to virtually visit the residents in terms of mobility, timing, and purpose. For example, Life Enrichment Coordinator Natasha (private LTC home) described how staff respected a family caregiver who would like to move around over the robot.

*“We have a resident, Ted, whose daughter calls in, she probably would not want it (the robot) to be mounted, because she likes to drive to the doorway and see where he is and she drives it up to him. She just really thinks it (the robot) is a good fit for her and her dad.”*

Other anecdotes originated at two different partner sites elaborated how family caregivers could virtually visit their loved ones over the robot without the limitation of timing and situation.

*“I haven't had to do too much with the family members at all. They just call whenever they want. The only concern they brought forward is when they were calling and the robot was covered, and that was mostly in the beginning. I think once staff has become more comfortable - they are not putting the covers on. Very rarely do I have to do anything with the family.”* (Courtney, Clinical Operations Supervisor, Ocean View)

*“We have used the telerobot with somebody who was in the end-of-life situation where family members couldn't come in because of COVID. The daughter was ready to give birth, so she did not want to come into the hospital [to visit her father in person] because she was in a very vulnerable situation. So just to protect her and the family, we offered them the telerobot for them to be with their loved one as much as possible, although it be just virtually. I found it very interesting because the daughter was at home, and she was just doing her daily living stuff – cooking and cleaning, etcetera. But [through the robot] she could see her father in our hospital room here. I feel just the voices and the sounds of home might have been very comforting for her father that was here.”* (Ashley, Patient Care Coordinator, Hospital)

#### Barriers: Insufficient resources and structural supports

Overall, care team leaders and members reported how limited resources in infrastructure, manpower, technology and physical environment that hindered and even frustrated them in using robots.

##### Wi-Fi issues

One of the biggest barriers raised by staff across care sites is a weak and unstable Wi-Fi connection. This has been mentioned by participants from the two focus groups and four individual interviews we conducted. Care leaders and members from three care sites, spanning the public hospital, public LTC and non-profit LTC home reported how poor wi-fi connection impacted social connection that ought to be facilitated by robots and frustrated staff to use the robot more. Below are two exemplary sharing from one of the interviews and one of the focus groups.

*“I think the only challenge [in using the robot] I would say is the disconnection. We had many conversations interrupted due to our Internet.”* (Bridgette, Clinical Operations Supervisor, Garden View)

Focus group conducted at Ocean View:

Courtney (Clinical Operations Educator): *“The Wi-Fi connectivity is a resource that is needed for successful implementation [of robots]. It is not great in some locations; in one of the rooms, it’s really good and then in another one, it comes and goes. So, when family members are calling, that sometimes leads to frustration.”*

Sarah (Clinical Educator): *“I can echo what Courtney is saying in terms of the Wi-Fi connection. We are happy to support, but sometimes it can be frustrating to the resident if they had an appointment (i.e., scheduled call from families over the robot) booked but it [the robot] is not working out.”*

##### Challenges in human resources

As mentioned earlier, our partner care sites sometimes experience staffing crises varying in extent, which leads to difficulties for staff to receive training about robots and to implement robots in their care delivery.

Insufficient staffing could also obstruct the team from using the robots in the long-term, as activities associated with maintenance, troubleshooting, and coordination of robots require staff’s attention. For example, when asked whether the care team would need ongoing technical support for the long-term implementation of robots, two participants from different LTC homes gave similar answers:

*“Yes, absolutely, especially with connectivity and technical issues.”* (Alex, Clinical Operations Educator, Ocean View)

*“I think it (technical support) has to be ongoing.”* (Bridgette, Clinical Operations Educator, Garden View)

In addition, a short-staffed care team could face difficulties in scheduling call-in with families due to robots being shared between residents at different rooms.

*“One challenge that I did come across was the timing. I know some passes for family members [last] for 2 to 3 hours. A lot of time it (scheduled calling time) is on the calendar but sometimes it gets missed – we get calls [from families]…Otherwise, its good.”* (Jasmin, TCW, Public Hospital)

A staffer reported an unexpected situation due to lack of coordination concerning a family call and concurrent care delivery for the same resident:

*“We had an incident where staff is just walking in and the robot moves. The families was trying to set it up (call through the robot) like a business call but it almost caused a staff member to stumble. Because the staff has no idea. It (the robot) was controlled by the family.”* (Bridgette, Clinical Operations Educator, Garden View)

##### Lack of technical support

Staff from different sites called for resources to support them as they are using the robot, in terms of troubleshooting and ongoing training for staff and families:

*“Staff are more than happy to help if the support and resources are there and organized for them. When they (the robots) get knocked off, maybe we should have a central number to call instead of waiting for one of the people who are the main users to go and find out [about technical problems concerning the robot].”* (Bridgette, Clinical Operations Educator, Garden View)

*“[We need] more hands-on training for the staff so they are comfortable. I think a lot of times when it's not docked or when it's not charged or if there's any technical support needed, the staff [are] still not very familiar [with handling] the robot issues. We need more hands-on and more continued training.”* (Lisa, Clinical Operations Supervisor, Garden View)

##### Limited physical space

Staff elucidated how a limited physical space discouraged them from using the robot. Two participant described unexpected situations, in which the robot was perceived as a safety hazard for residents and staff:

*“It happened once that the family was trying to drive it (the robot) and the staff was coming in the way. So, it was also a safety concern and better to have it stationary.”* (Bridgette, Clinical Operations Supervisor, Garden View)

*“The [physical] environment is a little bit tight, especially with residents who use wheelchairs, so I try to make sure it (the robot) is in a corner where it is safe for the resident and also for staff so that it is not a tripping hazard.”* (Sarah, Clinical Educator, Ocean View)

Other participants expressed their frustration about the incompatibility of placing a robot in a small area with the mobility needs and patterns of residents and staff, leading to an increased chance of a robot being knocked over:

*“Depending on where the robot is placed, there’s always a possibility that it could be knocked out of his docking station by either the staff or the resident when they pass by because of the tight corners. Those [possibilities] are what we observed.”* (Courtney, Clinical Operations Supervisor, Ocean View)

*“I think the design of the robot is a bit tricky, given the shared spaces. If it was a private room, it would be less challenging because you would have a designated area [to place the robot]. But for shared rooms, the space is so small. It is bound to be knocked off.”* (Bridgette, Clinical Operations Supervisor, Garden View)

Finally, some staff shared their observation on how shared rooms diminished users’ experiences with the robot, including their raised concern on privacy for residents and families.

*“Privacy is a thing because of the shared rooms. You have to be mindful of your roommate, for example, when the roommate has loud TV on, or music. One of the concerns from one of the families was that they are trying to talk to their loved ones through the robot. They (the families) were having a difficult time because their loved ones had difficulty with hearing because of the background noise.”* (Courtney, Clinical Operations Supervisor, Ocean View)

## Discussion

Our results indicated that structural and environmental factors are determinants that impacted staff’s usage of robot. In order to support residents who require comprehensive care delivery [28], care delivery staff require support and resources at multiple levels to increase their implementation of technology such as telecommunication robots. Our findings stressed the urgency to provide comprehensive structural support to care teams and homes. Further, as staff’s descriptions made references to unique organizational cultures, strengths, and challenges at each respective site, future resource allocation ought to be tailored to address such organization-specific preferences and needs.

In this study, staff indicated facilitators that help them implement robots in their care work. These factors were predominantly relational and cultural in nature, for example positivity in leadership, teamwork, and staff-family relationships. Barriers that were identified spanned structural, infrastructural, and technical factors that could not be easily overcome by individual staff members or care teams.

### Training and technical support to uplift existing strengths in care teams

Staff in our study were generally committed to delivering person-centred care to residents and open to learning from the research team or their peers. Most of the staff agreed that the implementation of robots was aligned with their care goals, and that they were willing to use robots to achieve said goals. This corresponds well with prior findings reported by Nielson et al. [29]. We argue that to achieve long-term or larger-scale implementation of telerobots, training and technical support needs to be sustainably available to staff. We also call for continuous acknowledgement and promotion of their successful experiences in technology usage, which was in line with an argument made by Wong et al. [30]. Future research could explore strategies concerning staff training, staff engagement, and the provision of support tools, as well as evaluate the effect of such strategies in the identified problem contexts.

### Under-resourced geriatric care sites

The existence of environmental and organizational barriers in the implementation of robots in care highlight deeper, longstanding challenges faced by geriatric care institutions across Canada. These challenges relate to shortcomings in human resources, infrastructure (e.g., Wi-Fi), technology, partnership with research teams, and physical environments [31–34].

With regard to human resources, our results indicated a paradox. On the one hand, the robot provided a chance to reduce staff’s workloads in the context of supporting residents’ personal communication. On the other hand, existing staffing crises undermined teams’ abilities to utilize the robot. Maintenance and troubleshooting of the robot require additional work from staff that adds to their existing routine. Personnel in understaffed units are often overloaded and vulnerable to issues like burnout [35–37]. These work experiences could lead to low job satisfaction and high staff turnover [38], which could undermine efforts to train staff in the handling of care robots. Thus, to ensure ongoing implementation of such robots, it would be necessary to overall improve human resources in LTC and hospitals. For example, human resources in these care organizations could be enhanced by having sufficient numbers of not only licensed care professionals, but also experts from various fields to improve the overall function of care sites, including fostering the interdisciplinary and multidisciplinary teamwork, providing technical supports, enhancing care sites’ partnerships with research teams, and supporting staff training, skill acquisition, and human resources on a long-term basis. In addition to human resources in LTC and hospital settings, future studies could investigate the cost-effectiveness of implementing such robot, and compare its cost-effectiveness with other robots and similar technology.

Regarding Wi-Fi, our data suggest that staff with access to reliable Wi-Fi are more likely to experience benefits from using the robot for residents, families, and themselves. Staff with positive experiences tend to use the robot more, which generates more positive impact on multiple stakeholders at different dimensions, such as the well-being of residents, improved family-staff relationships, reduced staff workload, and better staff work experiences. By contrast, staff were more frustrated when implementing robots at sites with unstable or slow Internet connection. They could not gain the abovementioned benefits but were obligated to spend more time on troubleshooting and coordination.

Another example of how resourcefulness in LTC homes determined the robot’s implementation is mobility. The size and layout of the physical space in which robots were used shaped staff’s usage of them. Homes and rooms with sufficient space, a well-designed layout, inviting atmosphere, and supportive staff were better positioned for families to use and move around robots. Driving the robot around helped families to identify new topics to converse with residents about and see the environment in which the residents are living. These shared experiences may improve the residents’ well-being, increase the transparency in the care home, and enhance the trust between staff and family. Conversely, nursing homes with limited space, narrow hallways, shared bedrooms, and overloaded staff were more likely to keep the robot mounted in place. Thus, future studies should explore how the mobility of telerobots impacts usage outcomes and which strategies could be used to overcome mobility restrictions.

Lastly, staff believed that changes to the robots’ design would help to better address stakeholder needs and environmental limitations. The specific facilitators and barriers identified align well with those referenced in related research. On the one hand, robot usage was facilitated by making the robot being easy to implement, which decreased staff’s involvement in setting up resident-family communications [12], and allowing staff and families to jointly work towards resident well-being [39]. On the other hand, staff in our study agreed with others who suggest that the robots’ audio may present challenges to some users [39, 40]. Our findings suggested that to improve the robots’ usage in LTC homes and hospitals, key partners should be involved when designing, testing, and implementing new versions of this and similar robots. These stakeholders include residents, family caregivers, and staff. In particular, the technology is suggested to embrace the personhood of residents and be user-friendly for formal caregivers, which is consistent with the argument by Wilson and Small (2020) [28]. Further, future studies are suggested to investigate on positive experiences and challenges of using this and similar robots from the views of residents/patients with dementia, family caregivers and care staff.

Our study provided a platform for staff’s perspectives on facilitators and barriers concerning the implementation of robots in dementia care sites. Our data provide a relatively strong representation of workforce in urban areas of British Columbia. The longitudinal data present the dynamics in the implementation of the robot as the study progressed. For example, we experienced differences in staff comments before and after training, and we identified more angles and facets in our understanding of the participants’ experiences as time went by. Our efforts in staff engagement added knowledge to an underexplored area of training and supporting staff to increase their robot usage in their care delivery for older adults with dementia. The training and supporting strategies tailored to staff’s needs also benefited the research team due to a promoted staff’s ownership in the study and staff’s willingness to share data with us, enhancing the quality of our data collected.

Two research assistants served in partnered care homes during the study period, which allowed us to gain rich contextual information and to collaborate with the care team more efficiently.

### Limitations

It is plausible that some staff members may be reluctant to share perceived barriers about their own workplace. Staff participants who had a positive experience with robots chose to participate in our interviews. Two co-authors worked as staff champions in partnered care sites, which may impact their perception to the robot. It is likely that they are generally more positive towards robots compared with other staff members who are not involved in research. For example, they may know more about the various functions and features of the robot and maybe be more active in using robots in different scenarios tailored to the environment and the resources in the care home they work at. Nonetheless, interviews with staff participants did reflect a range of both positive and negative views and offered insights for future research on the implementations of telerobots and similar technologies. Potential power dynamics may exist in our focus groups, but its impact was considered low by our research team. One out of four groups involved 8 participants from diverse professional backgrounds, however their care team lead did not participate in the focus group; we conducted the individual interview to the care lead separately, after the focus group. For the rest three focus groups, the focus group participants are staff leaders with equal authoritative powers, though they were from different training backgrounds.

Another limitation is that this research only focused on a specific population for the use of the robots – dementia LTC homes and hospitals. In a related vein, this research was based in an urban metropolitan city. It is possible that robot uses or staff experiences related to robot uses would look different in other care environments, with respect to conditions other than dementia, or in rural settings (where in-person visits may be harder to coordinate). Additionally, only staff who are fluent in English were included in this study, and our robot and training manual are limited to English.

### Conclusion

This study offered insights concerning facilitators and barriers to the implementation of telepresence robots in geriatric institutional care settings, as perceived by staff members. Our findings underscore the crucial role of care staff in hospital and LTC homes in facilitating interactions between residents, families, and robots. Tailored training and support are key to innovation adoption. Structural support is vital at individual and organizational levels to overcome barriers and foster adoption. Future research should explore staff and family caregivers' perspectives and strategies for providing support across diverse care settings.

### Supplementary Information

**Additional file 1: Appendix 1.** COREQ Checklist. **Appendix 2a.** Telepresence Robot User Instruction. **Appendix 2b.** Unbox Telerobot by Dr Jim Mann

## Data Availability

We do not have any research data outside the submitted manuscript file.
