# Supplementary material for: Working with a robot in hospital and long-term care homes: staff experience
Source: BMC Nurs. 2024 May 8;23:317. doi: 10.1186/s12912-024-01983-0 (PMC11080152; doi:10.1186/s12912-024-01983-0)
Supplement: Supplementary file 1 — Additional file 1: Appendix 1. COREQ Checklist. Appendix 2a. Telepresence Robot User Instruction. Appendix 2b. Unbox Telerobot by Dr Jim Mann [file 12912_2024_1983_MOESM1_ESM.docx]

**Appendices**

Appendix 1 – COREQ Checklist


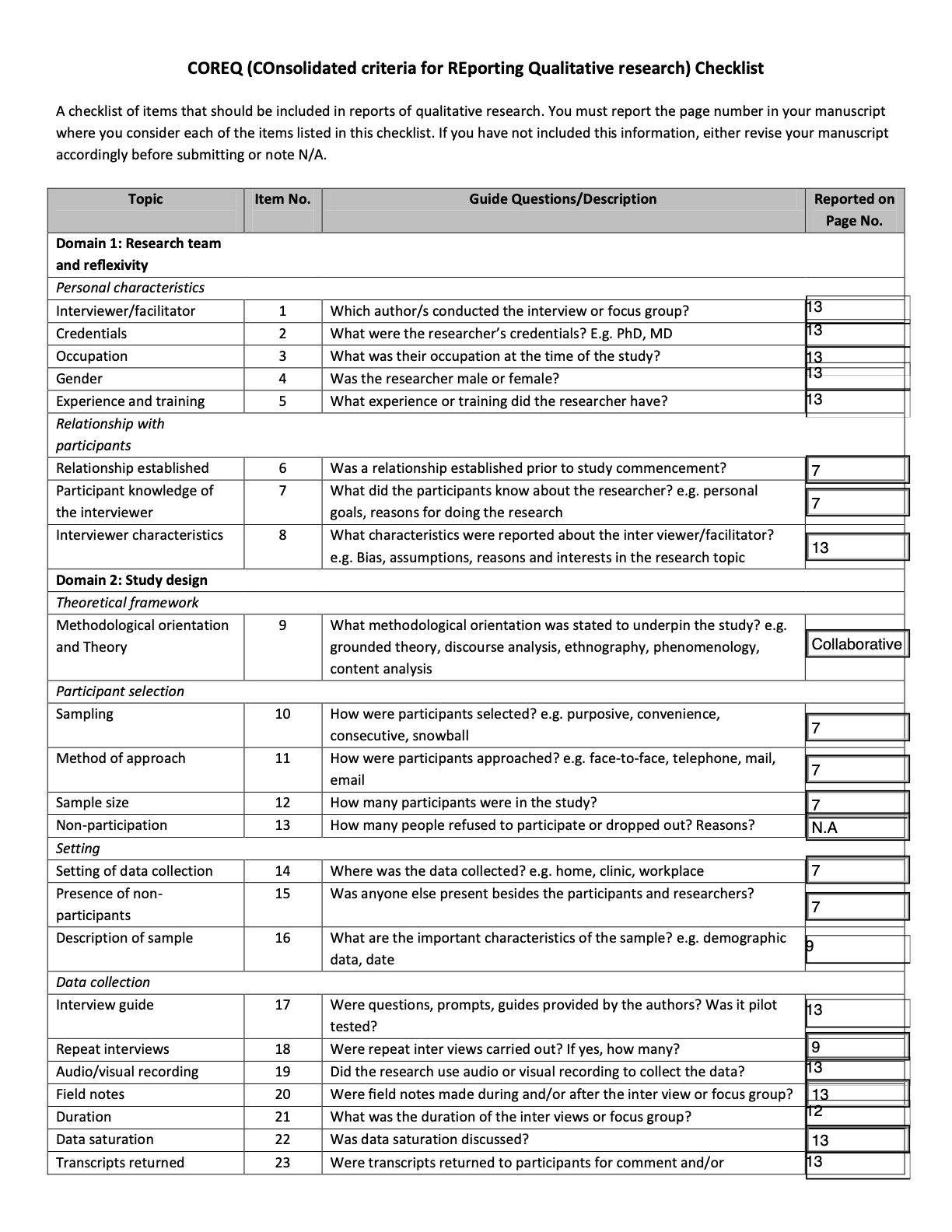


Collaborative action research


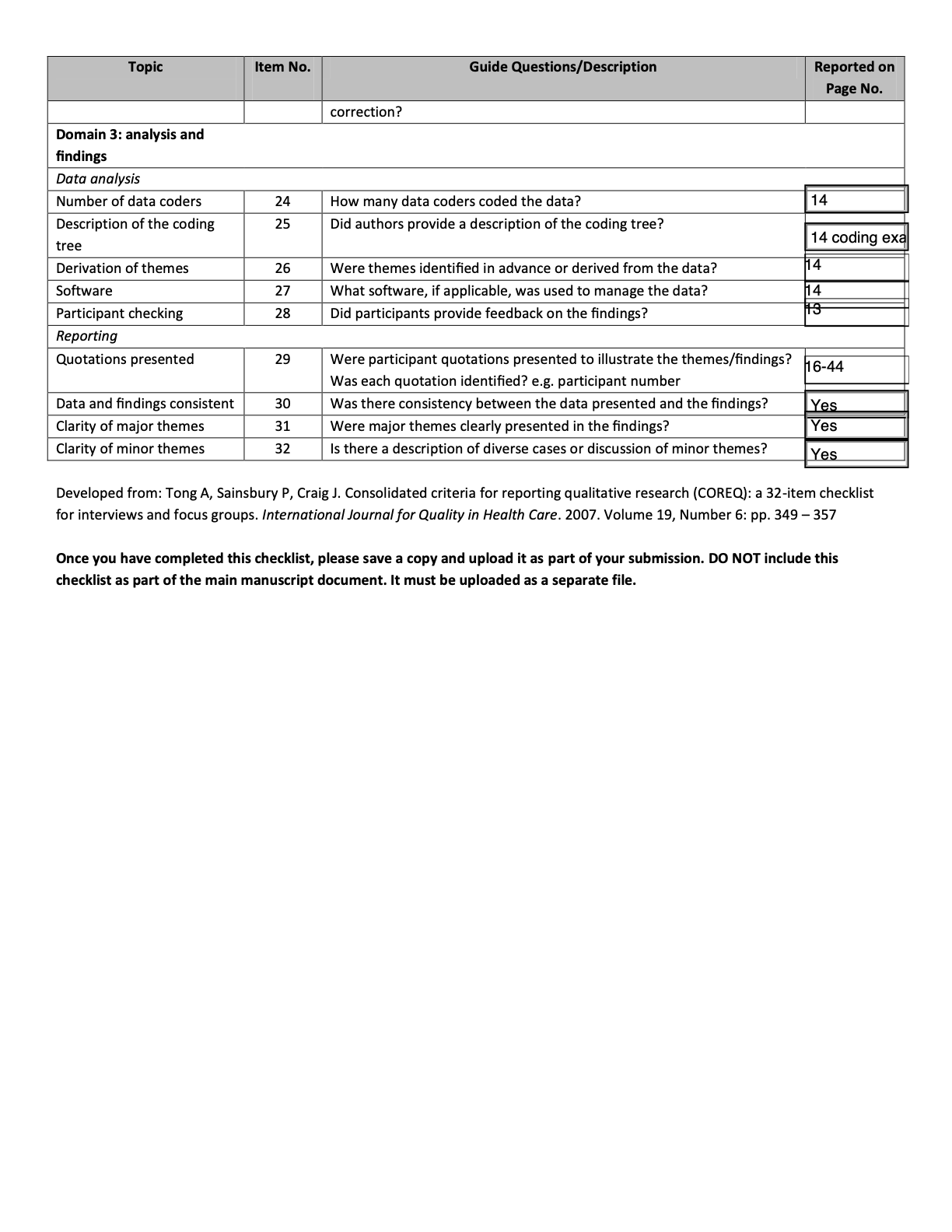


14 coding examples

Appendix 2a – [Telepresence Robot User Instruction](https://www.youtube.com/watch?v=Hayc3z9VoKQ&t=20s)

[
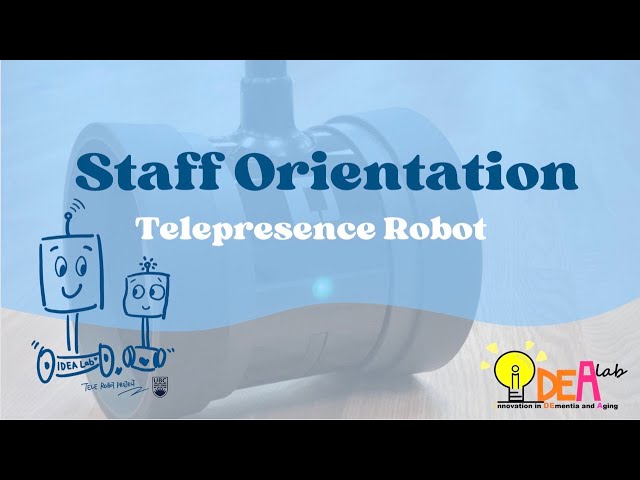
](https://www.youtube.com/watch?v=Hayc3z9VoKQ&t=20s)

Appendix 2b – [Unbox Telerobot by Dr Jim Mann](https://www.youtube.com/watch?v=8KYZKF5vXoA&t=43s)

[
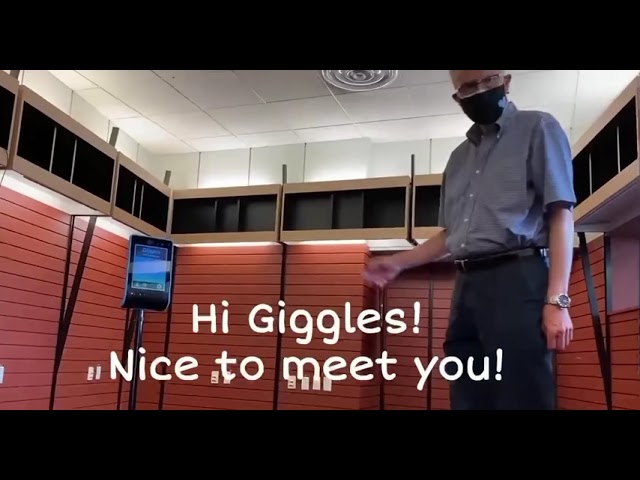
](https://www.youtube.com/watch?v=8KYZKF5vXoA&t=43s)
